# Elosulfase alfa in the treatment of mucopolysaccharidosis type IVA: insights from the first managed access agreement

**DOI:** 10.1186/s13023-021-01876-4

**Published:** 2021-09-25

**Authors:** Bob Stevens, Tom Kenny, Sophie Thomas, Alexandra Morrison, James Jarrett, Mohit Jain

**Affiliations:** 1 The MPS Society, Amersham, Buckinghamshire UK; 2 Rare Disease Research Partners, Amersham, Buckinghamshire UK; 3 BioMarin International Ltd., London, UK; 4 BioMarin Europe Ltd., 10 Bloomsbury Way, London, WC1A 2SL UK

**Keywords:** Managed access agreement, Mucopolysaccharidosis type IVA, Morquio A, Elosulfase alfa

## Abstract

Managed access agreements provide a crucial mechanism whereby real-world data can be collected systematically to reduce uncertainty around available clinical and economic data, whilst providing the opportunity to identify patient sub-populations who are most likely to benefit from a new treatment. This manuscript aims to share learnings from the first managed access agreement, which was initiated following positive conditional approval in 2015 from the National Institute for Health and Care Excellence (NICE) for elosulfase alfa, an enzyme replacement therapy for the treatment of mucopolysaccharidosis type IVA (MPS IVA). This managed access agreement enabled the collection of comprehensive real-world data for patients with MPS IVA, with results demonstrating that patients starting elosulfase alfa treatment showed gains similar to those seen in the pivotal trial for outcomes including endurance, respiratory and cardiac function, pain, quality of life measures and urinary keratan sulfate levels. In addition, former trial patients continued to see benefits in both clinical assessments and quality of life/activities of daily living nine years after beginning treatment. Key strengths of the process included recruitment of a high proportion of MPS IVA patients treated in England (72/89 known eligible patients) with a wide range of ages (2–58 years). Participation of a patient organisation (the MPS society) ensured that the patient voice was present throughout the process, whilst a contract research organisation (Rare Disease Research Partners) ensured that patients were represented when interpreting agreement criteria and during patient assessment meetings. Longer-term follow-up will be required for several MPS IVA outcomes (e.g. skeletal measures) to further reduce uncertainty, and continued follow-up of patients who had stopped treatment was found to be challenging. The burden associated with this managed access agreement was found to be high for patients, physicians, patient organisations, NHS England and the manufacturer, therefore costs and benefits of future agreements should be considered carefully before initiation. Through evaluation of the strengths and limitations of this process, it is hoped that learnings from this managed access agreement can be used to inform future agreements.

**Supplementary Information:**

The online version contains supplementary material available at 10.1186/s13023-021-01876-4.

## Introduction

Highly specialised technology (HST) evaluations are conducted by the National Institute for Health and Care Excellence (NICE) to make recommendations on the use of medicines and treatments within the National Health Service (NHS) in England for very rare conditions [1]. The HST process was established in recognition of the fact that orphan treatments are rarely cost-effective at thresholds applied to single and multiple technology appraisals (STAs and MTAs) [2, 3]. The small patient populations associated with rare conditions can often lead to high uncertainty due to a paucity of natural history, resource use and quality of life data [4]. Subsequently, insufficient evidence and high unit costs often prevent orphan drugs from meeting health technology assessment (HTA) cost-effective requirements, leading to restricted access to potentially life-changing treatments for patients with rare diseases [5].

To address these challenges, the HST programme uses a wider evaluation framework than STAs or MTAs, taking into consideration the nature of the condition in question and the technology’s perceived value for money. It also takes into account the expected impact on both direct and indirect health benefits, including costs to specialised and personal social services, as well as costs incurred outside of the NHS [6]. In 2017, NICE introduced a £100,000 willingness-to-pay (WTP) threshold, for HSTs that deliver ≥ 10 quality-adjusted life years (QALYs) per patient over their lifetime, with a QALY modifier allowing this to rise to £300,000 for ≥ 30 QALYs gained over a patient’s lifetime [7].

However, despite the additional criteria considered as part of the HST appraisal process, further data may still be required to minimise uncertainty in the reimbursement process. Subsequently, products which receive a conditional approval in the HST programme may be required to generate further evidence through a managed access agreement (MAA) in order for reimbursement to be considered in the long term [7].

The main objective of most MAAs is to help to reduce uncertainty (particularly around clinical effectiveness) through systematic collection of real-world data following receipt of HTA recommendations over a limited time period, and therefore reduce risk to payers investing in technologies which may subsequently prove not to be cost-effective [7]. Study outcomes may be different or in addition to clinical trial outcomes, but should be patient-relevant and targeted to reduce the uncertainties identified in the appraisal process [7], therefore it is vital that criteria for the MAA are aligned between participating stakeholders, including NICE, NHS England, participating physicians and patient groups.

One key area of uncertainty lies in the identification of patient sub-populations, and their subsequent response to the treatment, which can be difficult to ascertain during clinical trials, particularly in rare diseases where patient populations can be limited and presentation can be heterogeneous. It is therefore crucial to identify the most appropriate sub-population of patients who are most likely to gain benefit from an HST (e.g. patients with severe forms of the disease) in a real-world setting. MAAs can be used to reduce uncertainty in groups where studies have been limited or there is a question of value [8].

**Fig. 1:**
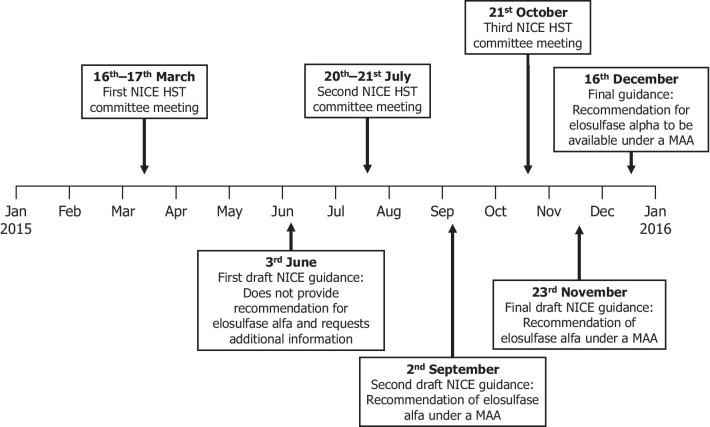
NICE guidance timeline prior to MAA initiation. HST, Highly specialised technology; MAA, managed access agreement; NICE, National Institute for Health and Care Excellence

MAAs have been used in STAs for some time, particularly through the Cancer Drugs Fund [9], however, relatively few interventions have been appraised by the HST process and fewer still through a MAA initiated in response to HST feedback [1]. The first instance of this occurred in December 2015, when elosulfase alfa received a positive conditional recommendation from NICE for treating mucopolysaccharidosis type IVA (MPS IVA or Morquio A) according to the conditions of a MAA (Fig. 1) [10]. MPS IVA is a progressive, ultra-rare genetic condition which, when left untreated, results in significant multisystemic morbidities and early mortality [11, 12]. Currently, the enzyme replacement therapy known as elosulfase alfa is the only disease-modifying treatment available for this disease [13]. NICE had requested the initiation of MAA on the grounds that it did not consider the cost of elosulfase alfa fully justified and could not ascertain whether the benefits of treatment measured in short-term clinical trials would, on average, be associated with gains in longevity and persisting benefits in those outcomes that are important to patients.{NICE, 2015 #24} The MAA was designed to address concerns raised by the NICE Committee in their evaluation and involved multiple stakeholders, including a representative of the treating physicians, a patient organisation (MPS Society), a contract research organisation (Rare Disease Research Partners; involved in the collection of patient-reported outcome data), the manufacturer (BioMarin International Ltd), NICE, NHS England, and crucially participating patients, who were considered to be key informed partners throughout the course of the MAA. At the time of publication, an extension to the MAA had been agreed between NICE, NHS England and the manufacturer following expiration of the original MAA terms (as of December 2020), and discussions with NICE regarding recommendation of elosulfase alfa were ongoing.

This article aims to share insights from this multi-stakeholder MAA based on our experience to date, evaluating the strengths and limitations of the programme from the perspective of a patient organisation, the contract research organisation and manufacturer.

## Methods

### Patient eligibility

In order to receive treatment with elosulfase alfa as part of the MAA, patients were required to sign up, have a confirmed diagnosis of MPS IVA as per the diagnosis criteria recommended in Wood et al. (2012) and comply with the associated monitoring criteria, including attendance at a clinic three times a year for assessment [14]. According to the MAA, treatment with elosulfase alfa would be ceased if the patient was non-compliant with assessments for continued therapy, was unable to tolerate infusions or failed to meet pre-specified monitoring criteria (two different sets of criteria were developed, distinguishing between patients who were, at the time, new to treatment [naïve responders] and those currently receiving treatment). Patients deemed to be ineligible for further treatment with elosulfase alfa will continue to be monitored for disease evolution and clinical outcomes and supported with other clinical measures in line with UK standard of practice [15]. Full eligibility criteria are detailed in the Additional file 1.

### Data collection and monitoring

Patients were required to attend medical assessments three times a year (additional information regarding the schedule of outcome assessments is included in the MAA) [16]. Clinical outcomes were assessed by the treating physician, and patient-reported outcome (PRO) tools were administered and assessed by Rare Disease Research Partners, acting on behalf of the MPS society. Each patient was assessed in the context of clinical and quality of life data, including endurance, respiratory and cardiac function, pain, quality of life, activities of daily living, depression and urinary keratan sulfate (uKS) levels. It should be noted that the MAA did not track safety information, except monitoring intolerance to treatment and antibody titres [17]. For the latest published safety information on the global population, the label should be consulted [17, 18].

Physicians and the contract research organisation (on behalf of the MPS society) provided interpretation and additional details, especially where a patient’s treatment response was considered to be borderline. Reasonable adjustments were made for patients who were unable to comply with the assessments. In these cases, a patient’s stop criteria were determined by individual agreement between the treating physician and NHS England. For example, if there were mitigating circumstances (e.g. surgery, illness) and a patient could not complete an assessment, the patient would be deferred until the next assessment. For patients who were < 5 years of age, stop criteria were not applied. For patients who had other cognitive impairments prohibiting completion of certain tests, clinicians were expected to make all possible efforts to gather as much information as possible. If a patient was deemed ineligible for further treatment with elosulfase alfa, patients could appeal if they felt the assessments were incorrectly performed. Each patient who was being assessed was considered to be either a pass, a fail, or a deferment.

Full monitoring criteria are described in the Additional file 1. A distinction was made in the monitoring criteria between naïve patients and patients who were receiving treatment: the latter were defined as clinical trial patients, patients otherwise already receiving treatment, and patients who started on treatment during the term of the MAA and have been receiving treatment for over 12 months. As part of the MAA, patients could voluntarily enrol into a 10-year disease registry (the Morquio A Registry Study [MARS]) for monitoring purposes [19].

All patients provided informed consent to take part in the MAA, to have their demographic and clinical data collected by their treating clinician and to have their PRO data collected by the contract research organisation. Data collected during the term of the MAA were owned by BioMarin but shared between the signatories of the MAA (excluding the MPS Society in light of restrictions associated with data confidentiality) for the purposes of annual assessments and the analysis of the final data set at the end of the MAA period. The MAA was formally reviewed by all signatories in the third year after its initiation, when it was agreed to continue without adjustment. Informal process reviews were also held after each assessment meeting with representatives from NHS England, NICE, the contract organisation and clinicians to identify and resolve any procedural issues. Other stakeholders (the MPS society and BioMarin) joined these process review meetings but did not attend patient assessment meetings to preserve patient confidentiality.

### Exit strategy

If, at the end of the 5-year MAA, elosulfase alfa is not recommended by NICE, NHS England funding for elosulfase alfa will cease to be available for all patients and treatment will be ceased (cessation will be managed by BioMarin and NHS England to ensure it occurs in a controlled manner). On the other hand, if the treatment is recommended, further funding from NHS England will be conditional on the agreement of commercial terms between NHS England and BioMarin. These potential eventualities were highlighted to patients ahead of participation in the trial to ensure they were able to make an informed decision.

## Learnings from the managed access agreement

As the first MAA initiated in response to NICE HST feedback, it is important to critically reflect on the elosulfase alfa MAA and share relevant learnings. Despite the recent development of formal guidance from NICE on the use of MAAs [1], best practice approaches for MAAs in rare diseases are still under debate, and subsequently the use of precedents and shared learnings may prove to be crucial for future agreements of this kind [8]. By sharing our insights from this process, we hope to inform the community on the strengths and challenges associated with the practical application of this methodology and, ultimately, to guide future agreements of this nature.

### Strengths of the MAA

Data were collected as part of this MAA with the aim of resolving uncertainty around the efficacy and safety of elosulfase alfa treatment in patients with MPS IVA, with results confirming similar trends to those seen in the pivotal trial, including improved or sustained endurance (as determined by the 6-min walk test or the 25-foot ambulation test) and respiratory function (as measured by FVC or FEV-1 tests), among other endpoints [20–27]. Furthermore, no patients in the MAA stopped treatment due to adverse reactions and antibody titres were in line with previously published reports [17, 23, 28]. These results also supported assumptions made in the initial health economic modelling results submitted as part of the NICE appraisal process [26, 27, 29]. Results from the MAA have reduced uncertainty around the cost effectiveness of elosulfase alfa, demonstrating an estimated QALY gain similar to the initial estimate provided to NICE ahead of MAA initiation. However, it should be noted that at the time of publication, the expected QALY gain and cost effectiveness of elosulfase alfa were undergoing evaluation through the NICE appraisal process.

This MAA also enabled the collection of comprehensive real-world data for patients with MPS IVA, providing crucial evidence of patients’ response to treatment in the long-term, as well as a further understanding of activities of daily living and quality of life. In particular, this MAA allowed longer-term data to be collected for former participants of the pivotal elosulfase alfa clinical trials, some of whom have been on treatment for over nine years, and in many cases a maintenance of endurance and lung function was observed in this cohort [27]. Furthermore, patients who participated in the MAA were given the option to enrol in a 10-year MPS IVA disease registry, allowing for further generation of longer-term health and quality of life outcomes [19]. This will provide access to a substantial pool of data that can support the continued evaluation of existing and future treatments, as well as helping to support a better understanding of disease progression and management [30].

**Fig. 2:**
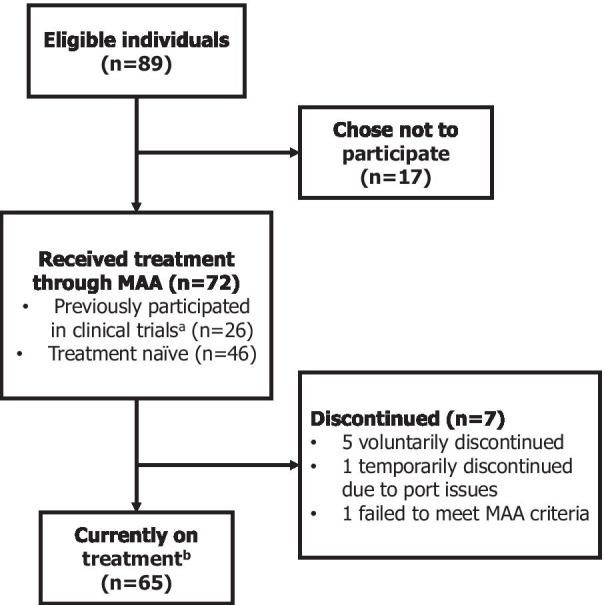
Patient disposition diagram. ^a^Previous elosulfase alfa clinical trials included NCT01415427, NCT01515956, NCT01697319, NCT00787995, NCT01242111, NCT01609062; ^b^As of 5th May 2020. MAA: managed access agreement

A high proportion of MPS IVA patients based in the England were recruited in this MAA with 72 of 89 eligible patients receiving elosulfase alfa as part of the scheme (Fig. 2) [31]. This cohort exhibited a mean treatment duration of 4.1 years in November 2019, and of those patients, 26 had previously participated in elosulfase alfa clinical trials, including 9 patients from the phase II MOR-002 study who had been receiving treatment for over nine years [32]. In context, 176 patients participated in the phase III trial from 17 countries [33]. The successful recruitment of a large number of MPS IVA patients was considered a key strength of the scheme, maximising both the number of patients able to access a novel treatment and the amount of data collected as part of the process. This highlights the importance of the networks developed by physicians and patient groups, enabling information surrounding the scheme to be communicated to eligible MPS IVA patients. The MAA also allowed patients with a wide range of ages to participate (2–58 years at the time of initiation of participation) [32], allowing data collection in a wider range of patient sub-populations than would typically be eligible in the clinical trial setting, where selection criteria for eligible participants are applied. Interestingly, no sub-populations with identifiable characteristics in this study were observed despite the wide range of ages of patients taking part.

The multi-disciplinary and multi-stakeholder approach to data collection and assessment of patients was a key strength of the scheme, which aimed to ensure a fair and holistic view of the patient’s progress on treatment. Critical to this, were the physicians and in particular the MPS Society, who ensured that the patient voice was heard throughout the process, as well as the contract research organisation (Rare Disease Research Partners) who ensured that the patient voice was presented during interpretation of MAA criteria and through attendance of patient assessment meetings. It has previously been observed that the research value of PROs may not be fully understood by patients, which can lead to a negative impact on PRO data collection [34]. The presence of a patient group representative may help to clarify this for the patients and thereby improve the quality of data collection. Consequently, in this MAA, PROs were administered and assessed by Rare Disease Research Partners, acting on behalf of the MPS society. Rare Disease Research Partners also collected additional patient testimonies to enable accurate presentation and interpretation of PRO data in the context of a patient’s current situation. Patient group representatives also helped to encourage patients to complete measures whilst providing support to those who faced challenges in providing a response, allowing improved collection of robust patient-relevant data.

One of the key strengths associated with MAAs in general is their provision of access to potentially life-changing technologies for patients where they may otherwise not have been recommended by an HTA body due to the levels of uncertainty and drug costs. A possible alternative in such circumstances would be the initiation of an additional clinical trial, if commercially possible, but this would likely lead to further access delays and a burdensome process for patients and carers.

### Limitations of the MAA

Despite the additional data collected during the course of this MAA, it should be noted that areas of uncertainty around the use of elosulfase alfa remain. During the NICE HST appraisal, it was acknowledged that it would take many years to assess the skeletal impact of elosulfase alfa, which was beyond the scope of the 5-year MAA [29]. Although skeletal impact can be monitored through measurement of growth, this was not considered to be an appropriated common clinical endpoint given some patients had stopped growing, and subsequently, growth was not measured as part of the MAA. However, it should be noted that the impact of elosulfase alfa on growth is being investigated as part of the ongoing MARS registry, building on previous research in which significant growth improvements in elosulfase alfa-treated patients were not observed [19, 35]. In addition, longer-term follow-up is required to investigate key outcomes such as cardiac and respiratory function, which are known to be the main causes of mortality in MPS IVA patients [12].

With respect to other clinical endpoints measured during the MAA, it should be acknowledged that uKS levels have not been proven to be correlated with clinical improvement and subsequently, were not used to monitor therapeutic efficacy [36, 37]; however, uKS is a rapid indicator of drug activity in the body and, as it is routinely used in clinical practice, was chosen as a marker of drug activity [38].

It is also important to acknowledge that conducting longer MAAs may not necessarily be the answer to improving patient access to novel treatments. Ultimately, reducing uncertainty can be both time and resource-intensive for patients, physicians and manufacturers, and can also delay treatment access to those ineligible to participate in an MAA. Thus, it is important to consider the costs and benefits when planning real-world data generation post-HTA appraisal. In the case of this MAA, the original pivotal trials had a placebo-controlled study length of 24 weeks [33], therefore it was felt that a 5-year MAA would provide a sizeable reduction in uncertainty for elosulfase alfa. However, it may be advisable for manufacturers to conduct a value of information analysis ahead of MAA initiation, i.e. as part of the HST process, in order to understand the value of additional data generation before making this commitment [39]. Manufacturers should also consider the logistical and ethical implications in the event that a treatment does not achieve reimbursement, whilst making any potential risks clear to patients at initiation of the scheme as described above.

When designing an MAA, it is also important to consider the likelihood of reducing uncertainty against the potential administrative burden placed on all stakeholders, with particular focus on that of the patient. Logistics surrounding the data collection process should be carefully considered in advance to avoid unnecessary confusion between stakeholders and/or any data errors which could result in a poorer outcome for a patient.

In addition, the assessment schedule should be designed to ensure accurate monitoring of key clinical variables, while minimising the impact on the patient and health care professionals. In this MAA, non-compliance was defined as missing > 3 infusions in any given 14-month period and, subsequently, patients and/or their carers were required to spend a significant time in hospitals on clinic visit days and attending 7 telephone appointments to assess PROs (although if this was not possible, alternative arrangements could be made). These requirements may have resulted in a considerable time burden for patients and/or their carers.

However, incorporating multiple chances to review and alter the processes associated with the MAA can potentially improve overall results and minimise the burden on patients and the health care system. For example, in the MAA described here, a review process resulted in both changes to the data collection process and a reduction of burden on patients and their families by reducing the number of clinic visits from three to two times per year [11, 40].

For those who discontinued elosulfase alfa therapy, patient follow-up was also found to be challenging. Following discontinuation, patients were less likely to attend the clinic for follow-up assessments or participate in PRO assessments, despite continual encouragement from the patient organisation. This resulted in an increased resource and time burden on the patient organisation and should be minimised where possible in future MAAs.

## Conclusions

Here we have reported insights from the first MAA initiated in response to NICE HST feedback, from the perspective of the patient organisation, contract research organisation and manufacturer involved in its initiation. This MAA set out to reduce the uncertainty around the short- and long-term effectiveness of elosulfase alfa for patients with MPS IVA whilst providing much-needed treatment access. The process of assessments revealed that patients starting treatment as part of the MAA showed gains similar to those seen in the pivotal trials [33], and that those former trial patients continued to see benefits in both clinical assessments and quality of life/activities of daily living after nine years [27]. Through evaluation of the strengths and limitations of this process, it is hoped that learnings from this MAA can be used to inform future agreements.

## Supplementary Information

**Additional file 1**. Eligibility and Monitoring Criteria.

## Data Availability

Not applicable.
