# Supplementary material for: Elosulfase alfa in the treatment of mucopolysaccharidosis type IVA: insights from the first managed access agreement
Source: Orphanet J Rare Dis. 2021 Sep 25;16:394. doi: 10.1186/s13023-021-01876-4 (PMC8467187; doi:10.1186/s13023-021-01876-4)
Supplement: Supplementary file 1 — Additional file 1. Eligibility and Monitoring Criteria. [file 13023_2021_1876_MOESM1_ESM.docx]

**Elosulfase Alfa in the Treatment of Mucopolysaccharidosis Type IVA: Insights from the First Managed Access Agreement**

Bob Stevens,^1^ Thomas Kenny,^2^ Sophie Thomas,^1^ Alexandra Morrison,^2^ James Jarrett,^3^ Mohit Jain^4^

^1^The MPS Society, Amersham, Buckinghamshire, UK; ^2^Rare Disease Research Partners, Amersham, Buckinghamshire, UK; ^3^BioMarin International Ltd., London, UK; ^4^BioMarin Europe Ltd., London, UK.

**Correspondence to:**

Mohit Jain

BioMarin Europe Ltd,

10 Bloomsbury Way,

London

WC1A 2SL

**Email address:** mjain@bmrn.com

**Short title:** Elosulfase Alfa Managed Access Agreement

**Funding:** BioMarin Pharmaceutical Inc

**Key words:** Managed access agreement, mucopolysaccharidosis type IVA, Morquio A, elosulfase alfa

**SUPPLEMENTARY APPENDIX**

**Eligibility Criteria**

- To receive treatment, patients must sign up to the Managed Access Patient Agreement.
- Patients are required to attend their clinics three times a year for assessment.
- Children under the age of 5 may not be able to complete all baseline and subsequent assessments. Clinically relevant assessments should be attempted at least once every 12 months until the age of 5, at which point all assessments become compulsory. There may also be other patients, i.e. those with cognitive impairments, who are unable to complete a full set of tests at appointed visits. In such cases, clinicians will be expected to make all possible and reasonable efforts to gather as much of the required data as possible.
- Elosulfase alfa will not be started if any of the following apply:
  - The patient is diagnosed with an additional progressive life limiting condition where treatment would not provide long-term benefit e.g. cancer or multiple sclerosis.
  - The patient has a lung capacity (forced vital capacity [FVC]) of less than 0.3 litres and requires ventilator assistance.
  - The patient is unwilling to comply with the associated monitoring criteria (all patients are required to attend their clinics three times a year for assessment.)

**Monitoring Criteria**

***Start Criteria***

- Patients must have a confirmed diagnosis of MPS IVA as per the diagnosis criteria recommended in Wood *et al* (2012).
- Patient must have a confirmed enzymatic test, elevated urinary keratan sulfate (uKS) and mutation analysis.
- A full set of patient baseline criteria must be obtained during the initial medical assessment prior to treatment commencing.
- Patients/parents will be expected to attend their clinic for assessment three times within any 14-month period.
- In the event of the patient being unable to maintain the above criteria, the implementation of the stop criteria will be discussed with the patient/parent.

***Stop Criteria***

Patients will become ineligible for further treatment where:

- The patient is non-compliant with assessments for continued therapy, where non-compliance is defined as fulfilling fewer than three attendances for assessment in any 14-month period.
- The patient fails to meet 4 of the 5 criteria as defined below for naïve responders and patients who are currently on treatment.
- The patient is unable to tolerate infusions due to infusion-related severe adverse events that cannot be resolved.

*Naïve responders (patients who have never received elosulfase alfa treatment)*

A responder following the first year of treatment for a treatment naïve patient will demonstrate at least four out of five of the following criteria otherwise they must cease treatment with enzyme replacement therapy:

- Improvement of 6-minute walk test (6MWT) or 25ft Ambulation Test of at least 10% improvement over baseline, or stabilisation after plateauing to a 10% improvement. Baseline will be a single 6MWT test performed according to American Thoracic Society guidelines and applied at a time the patient is in suitable condition that the test is not confounded by other health issues e.g. chest infection, cold etc. If a patient has had any minor surgery in the previous 3 months or major surgery in 6 months they will still take the test but it will be repeated. 6MWT will not be performed within 2 hours of respiratory function testing or any endurance assessments. The following will also be recorded in all patients over the age of 5 at both start and end of 6MWT: heart rate, oxygen saturation, respiratory rate and Borg scale. These values are required to support the validity and effort of 6MWT but only the total distance will be used for determining the stop point.
- Improvement in FVC or Forced Expiratory Volume (FEV)-1 measured with standard spirometry of 5% over baseline in the first year or stabilisation after the first year. Both are standard measures and the best values from 3 attempts will be used to determine the stop criterion but improvement in either FVC or FEV-1 will be sufficient as both measure different aspects of respiratory disease. Pulmonary Function Testing should not be done within 2 hours of endurance testing and if the patient is on any inhaler this should be used as appropriate. The other tests should be delayed if the patient is unwell (a respiratory rate higher than normal or a temperature greater than 38 degrees centigrade).
- Stabilisation defined as no adverse change in the numerical value in two of the following three measures:
  - the score of Quality of Life as measured by utility derived from EQ-5D-5L scores OR caregiver burden as measured by Mucopolysaccharidosis Health Assessment Questionnaire (MPS-HAQ) Caregiver Domain
  - Beck depression score
  - Adolescent Pediatric Pain Tool (APPT) or Brief Pain Investory (BPI) pain score depending on age
- Reduction from baseline in uKS of 20%
- Decline in ejection fraction of less than 10% from baseline as measured by echocardiogram.
- Patients will cease to quality for treatment if they miss more than 3 infusions in any 14 month period, excluding medical reasons for missing dosages.

*Patients who are currently on treatment*

Patients who are ‘currently on treatment’ are defined as: (i) clinical trial patients; (ii) patients otherwise already receiving treatment and have become a commissioning responsibility of NHS England; and (iii) patients who started on treatment during the term of the Managed Access Agreement and have been receiving treatment for over 12 months. To remain on treatment patients must fulfil four out of five of the response criteria:

- 6MWT and 25ft Ambulation Test remains 5% above baseline value at start of treatment with same limitations as for treatment naïve patients.
- FVC and FEV-1 remain 2% above baseline at start of treatment.
- uKS levels remain reduced at least 20% from baseline value.
- Stabilisation is defined as no adverse change in the numerical value in two of the following three measures:
  - the score of Quality of Life as measured by utility derived from EQ-5D-5L scores OR caregiver burden as measured by MPS-HAQ Caregiver Domain
  - Beck depression score
  - Adolescent Pediatric Pain Tool (APPT) or Brief Pain Investory (BPI) pain score depending on age
- Decline in ejection fraction of less than 10% from baseline as measured by annual echocardiogram.
- Patients will also cease to qualify for treatment if they have a lung function (FVC) of less than 0.3L and require ventilator assistance.
- Patients will cease to quality for treatment if they miss more than 3 infusions in any 14 month period, excluding medical reasons for missing dosages.
